# Retropharyngeal haematoma – an unusual bleeding site in an anticoagulated patient: a case report

**DOI:** 10.1186/1757-1626-1-294

**Published:** 2008-11-02

**Authors:** Seema Srivastava, Tarun Solanki

**Affiliations:** 1 Taunton and Somerset Foundation Trust, Musgrove Park, Hospital, Taunton, TA1 5DA, Somerset, UK

## Abstract

**Introduction:**

Anticoagulation is used widely for the primary prevention of embolic events in patients with atrial fibrillation. Bleeding is the most common complication with oral anticoagulation. We describe the case of a patient who developed a massive retropharyngeal haematoma after a fall. Whilst the retropharyngeal space is an uncommon site for bleeding complications, it is clinically important as the development of upper airway obstruction may be life threatening.

**Case Presentation:**

We present the case of an 85-year-old Caucasian woman on warfarin, who developed a massive retropharyngeal haematoma after a fall. She initially presented with pulmonary oedema and Type 2 respiratory failure. She was commenced on treatment for this with a good clinical response. She subsequently deteriorated, developing stridor and bruising to the neck. She was urgently intubated and ventilated. Computerized Tomography scan showed a massive retropharyngeal haematoma. The baseline International Normalized Ratio (INR) was 4.9. The patients was managed conservatively and treated with Vitamin K and Prothrombin Complex Concentrates (PCCs). The INR was rapidly corrected to 1.1 and the patient made a full recovery.

**Conclusion:**

Retropharyngeal haematoma should be considered in anticoagulated patients presenting with abrupt respiratory distress after minor head trauma. It can develop after minor traumatic events, such as falls. It can result in upper airway obstruction, which can be life threatening. Patients should be urgently assessed for intubation and ventilation. Computerized Tomography imaging of the neck and mediastinum is diagnostic. Correction of the International Normalized Ratio with Vitamin K and Prothrombin Complex Concentrates is essential. Management is mainly supportive. However, in very large haematomas surgical drainage may be considered.

## Background

Oral anticoagulation is commonly used in patients with atrial fibrillation for the prevention of embolic complications. Bleeding is the most common complication of this treatment. One study following 3958 patients on warfarin reported an incidence of bleeding requiring hospitalization of 2.6 per 100 patient years [1]. Although the retropharyngeal space is an uncommon site for bleeding complications, a haematoma here can expand almost unrestrictedly and rapidly lead to upper airway obstruction. We report the case of retropharyngeal haematoma in a patient on warfarin, as a result of a fall.

## Case presentation

**Figure 1:**
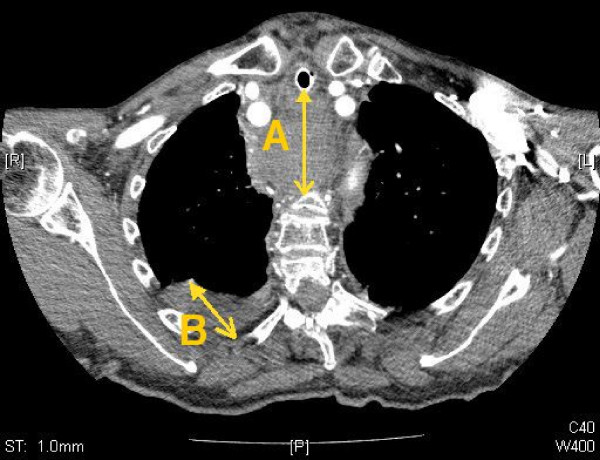
**Computerized Tomography of the chest; Computerized Tomography image of the thorax and mediastinum taken after the patient was intubated.** The image shows extensive retropharyngeal haematoma (A). There is also evidence of a right pleural effusion (B).

An 85 years old woman was brought to the emergency department after collapsing at home with breathing difficulties. On arrival she was dyspnoeic with oxygen saturation of 75% on room air and a respiratory rate of 40 per minute. She also had minor bruising to her forehead as a result of her fall. She had a past history of a porcine aortic valve replacement 17 years previously, hypertension and atrial fibrillation for which she was on warfarin. On examination she was tachycardic, hypertensive and her jugular venous pressure was elevated. She had coarse crackles throughout both lungs. Arterial blood gases on 15 litres oxygen showed severe type 2 respiratory failure with pH of 7.15, pC02 9.12, p02 10.22 and bicarbonate of 22.8 mmol/l. Laboratory results showed a haemoglobin of 11.9 g/dL and INR of 4.9. ECG showed atrial fibrillation, right bundle branch block and widespread ST depression. Findings on chest radiograph were compatible with pulmonary oedema. She was commenced on a nitrate infusion, intravenous diuretics and Bi-level Positive Airway Pressure ventilation. Her condition improved rapidly and the Bi-level Positive Airway Pressure ventilation was discontinued. An hour later her breathing deteriorated and she newly developed stridor. She was noted to have developed bruising of the neck. Urgent nasoendoscopy showed a normal larynx with a slight bulge narrowing the trachea anteriorly. She was immediately intubated and ventilated. An urgent computerized tomography (CT) scan of the neck showed a large retropharyngeal haematoma causing anterior compression of the airway, extending into the posterior mediastinum and terminating behind the left atrium. Evidence of pulmonary oedema and a right pleural effusion were also seen on CT. (Figure 1).

Intravenous Vitamin K at a dose of 5 mg and Prothrombin Complex Concentrates (PCCs) at a dose of 50 units per kilogram were given on diagnosis of the haematoma for the reversal of anticoagulation. The INR was 1.1 when repeated subsequently 2 hours later. Due to complications of a ventilation-related pneumonia the patient stayed in intensive care for 10 days after which she was extubated uneventfully. A decision was made not to recommence anticoagulation with warfarin due to the past history of recurrent injurious falls and the high risk of complications from future haemorrhagic events. The patient was commenced instead on aspirin and was discharged two weeks later to her own home.

## Discussion

Haemorrhagic complications from warfarin treatment account for 10.5% of all adverse drug reactions resulting in hospital admissions [2]. Bleeding is more likely to occur with an INR greater than 4.5 [3]. Older patients are more sensitive to the effects of warfarin and are more likely to have bleeding complications [4]. Haemorrhage related to anticoagulation is most commonly observed in the gastrointestinal tract, subcutaneous tissue and intracranially. Although there is limited data regarding bleeding risk for anticoagulated patients who fall, national consensus guidelines include recurrent or injurious falls as risk factors for haemorrhagic complications with warfarin [4]. There is no consensus on anticoagulation reversal for patients presenting with minor head trauma although one recent study suggests that all traumatic brain injured patients who are supratherapeutically anticoagulated should be considered for reversal of anticoagulation [5].

Retropharyngeal haematomas are uncommon but are a recognized complication of cervical spine trauma, great vessel trauma, foreign body ingestion, violent coughing or sneezing, retropharyngeal infections and iatrogenic injury after procedures such as internal jugular line insertion [6]. Eighteen cases of retropharyngeal haematoma secondary to oral anticoagulation have been previously reported [6]-[13]. Bleeding may be spontaneous or occur after minor trauma such as falls [8,12,13]. Haematomas in the retropharyngeal space can expand rapidly causing obstruction of the pharynx, larynx, oesophagus and trachea. Patients may present with a sore throat, hoarse voice, dysphagia, odynophagia, drooling, or overt stridor [6]. Tenderness, swelling or bruising of the neck may be evident. Lateral radiograph of the cervical spine may be useful in showing prevertebral soft tissue swelling and a chest radiograph may show a widened mediastinum if the haematoma is extensive [13].

Nasoendoscopy may show bulging of the posterior pharynx wall. Unstable patients with dyspnoea and hypoxia should be evaluated immediately and considered for intubation to protect the airway from obstruction. CT of the neck and mediastinum is diagnostic and will show the extent of haematoma and its proximity to other structures within the neck [14].

Most patients with retropharyngeal haematoma are managed conservatively. The haematoma will usually resolve with conservative management but can take several weeks [14]. In almost all of the previously published cases either vitamin K or a combination of either vitamin K and Fresh Frozen Plasma (FFP) or PCCs were used. British Committee for Standards in Haematology Guidelines recommend reversal of anticoagulation in major bleeding using factor concentrate and vitamin K [15]. PCCs are recommended in preference to FFP as complete and rapid reversal is more readily achieved, usually within 10–15 minutes, without the need for blood group typing and thawing before it can be used. Occasionally surgical drainage is required if the haematoma is very large or impeding mechanical ventilation. Late complications include non-resolving haematoma or abscess formation. There is little consensus in the literature regarding the use of steroids or prophylactic antibiotics in patients with retropharyngeal haematoma [6].

## Conclusion

Retropharyngeal haematoma should be considered in anticoagulated patients presenting with abrupt respiratory distress after minor head trauma. It can result in airway obstruction, which can be life threatening. Urgent assessment for intubation and ventilation is essential. Correction of the INR with vitamin K and Prothrombin Complex Concentrates is essential.

## Abbreviations

Mmol/l: Millimoles per litre; PH: Hydrogen Potential; pO2: Partial Pressure of Oxygen; pCO2: Partial Pressure of Carbon Dioxide; INR: International Normalized Ratio; ECG: Electrocardiogram; CT: Computerized Tomography; PCC: Prothrombin Complex Concentrates; FFP: Fresh Frozen Plasma.

## Competing interests

The authors declare that they have no competing interests.

## Authors' contributions

The authors were involved in the writing of the manuscript or patient clinical care. All authors read and approved the final manuscript.

## Consent

Written informed consent was obtained from the patient for publication of this case report and accompanying images. A copy of the written consent is available for review by the Editor-in-Chief of this journal.
